# International longitudinal registry of patients with atrial fibrillation and treated with rivaroxaban: RIVaroxaban Evaluation in Real life setting (RIVER)

**DOI:** 10.1186/s12959-019-0195-7

**Published:** 2019-04-25

**Authors:** Jan Beyer-Westendorf, A. John Camm, Keith A. A. Fox, Jean-Yves Le Heuzey, Sylvia Haas, Alexander G. G. Turpie, Saverio Virdone, Ajay K. Kakkar, for the RIVER Registry Investigators, Ajay K. Kakkar, A. John Camm, Jean-Yves Le Heuzey, Keith A. A. Fox, Jan Beyer-Westendorf, Sylvia Haas, Alexander G. G. Turpie, Karen S. Pieper, Gloria Kayani, Bernard J. Gersh, P. Hildebrandt, H. Dominguez, W. Comuth, L. Frost, D. S. Moller, H. Christensen, L. M. Bruun, A. Milhem, J. Gauthier, C. Mielot, S. Chanseaume, S. Chopra, A. Amlaiky, O. Tricot, V. Sierra, A. Dompnier, N. Zannad, A. Pinzani, A. Quatre, J. Mansourati, L. Fauchier, N. Badenco, E. Gandjbakhch, K. F. Chachoua, V. Malquarti, F. Pierron, F. Sacher, J. Taieb, J. M. Davy, E. Marijon, N. Lellouche, A. Leenhardt, A. Salem, I. Lesto, J. J. Muller, R. Garcia, J. P. Neau, J. B. Berneau, N. Schön, D. Gulba, K. F. Appel, J. Merke, J. Dshabrailov, C. Bauknecht, O. Scheuermann, T. Schröder, W. Jung, A. Kopf, J. Brachmann, M. Leschke, J. Taggeselle, M. Seige, T. Läßig, S. Appel, M. Schmiedl, K. Müller, G. U. Heinz, C. Axthelm, K. Eberhard, B. Hügl, T. Schwarz, U. Sechtem, A. Falanga, V. Rubino, L. Calo, W. Ageno, F. Massari, D. Imberti, L. Di Gennaro, F. Gaita, A. Margonato, G. Cannava, F. Capasso, I. Diemberger, F. Pelliccia, A. Cafolla, S. Bardari, L. Mattei, L. Ruocco, G. Boriani, D. Poli, S. Testa, C. Indolfi, R. Quintavalla, L. Mos, G. Ladyjanskaia, I. Aksoy, M. Van De Wetering, L. Theunissen, F. Den Hartog, R. Nijmeijer, R. Van De Wal, S. Reinders, M. Patterson, E. D. Melker, R. Troquay, J. Korecki, A. Szyszka, F. Diks, J. Sumis, J. Cygler, B. Miklaszewicz, E. Litwiejko-Pietrynczak, P. Napora, G. Drelich, T. Kawka-Urbanek, J. K. Wranicz, M. Mierzejewski, A. Drzewiecka, D. Wronska, I. Fares, J. Baska, K. Stania, W. Krzyzanowski, P. Miekus, M. Tyminski, D. Dronov, S. Zenin, E. Isaeva, A. Lopukhov, V. Yakusevich, D. Kuznetsov, T. Kameneva, E. Pokushalov, V. Karetnikova, I. Dik, I. Karpushina, D. Nikolin, A. Doletsky, A. Ardashev, A. Timofeeva, O. Miller, N. Lyamina, Y. Shubik, S. Boldueva, J. L. Blanco Coronado, C. Gonzalez Juanatey, E. Otero, D. Alonso, J. Torres Llergo, J. Gonzalez Lama, J. A. V. De Prada Tiffe, F. J. Garcia Seara, J. J. Gomez Doblas, J. A. Riancho, J. L. Clua-Espuny, J. Motero, V. I. Arrarte, D. Martin Raymondi, G. Isasti Aizpurua, F. Marin, J. A. Nieto, J. Fernandez Portales, P. Alvarez Garcia, I. Torstensson, B. Cederin, T. Kalm, U. Rosenqvist, J. Thulin, A. Hajimirsadeghi, M. Crisby, A. Manoj, A. Bakhai, A. Mistri, M. Krishnan, S. Kumar, S. Kirubakaran, H. Thomas, J. Camm, F. Ahmed, A. M. Ross, K. Barry, R. Stockwell, A. Broadley, M. Mamun, K. Chatterjee, J. Cooke, J. McCready, D. Dutta, K. John, P. Pandya, R. Howlett, P. Vinson, Lim, P. Foley, D. Bruce, A. Dixit, D. Broughton, J. Taylor, R. Schilling, K. Leon, K. Saeed, S. Shaheen, M. Tawfik, A. Mortadda, M. Seleem, M. S. I. Aly, G. Kazamel, M. Elbadry, S. Kamal, M. Hassan, M. Mostafa, M. E. S. Medhat, Ekhlas, R. Ghaleb, M. O. Taha, I. Daoud, H. Al Din, A. M. Imam, M. A. El Hameed, Helmy, M. Al-Murayeh, N. Akhtar, B. M. Matto, M. A. Ghani, O. A. Amoudi, M. M. Morsy, A. A. F. Bashir, Y. M. Al Hossni, B. Al Ghamdi, Zia-ul-Sabah, S. Mir, D. Dardir, A. Masswary, A. R. Al Shehri, A. Masswary, J. Iqbal, M. A. J. Almansori, C. G. Venkitachalam, J. Kurian, J. Rao, A. Aisheh, A. A. Albawab, B. Subbaraman, A. Amanat, K. J. Esfehani, R. Lochan, A. Bin Brek, B. Mittal, Y. Ghazi, M. Krishna, S. B. Tabatabaei, P. S. Thoppil, S. Nasim, S. El Khider Nour, P. Barros, A. P. Almeida, M. Andrade, B. Garbelini, T. L. Silvestrini, Á. R. Alves Junior, C. E. B. de Lima, A. Kormann, G. G. de Lima, C. Halperin, D. Salvadori Junior, A. F. Freitas, J. R. Gemelli, C. E. Ornelas, J. M. M. Dantas, J. L. Aziz, L. M. Backes, W. S. Barroso, M. S. Paiva, J. A. de Figueiredo Neto, F. R. dos Santos, J. A. de Lima Neto, R. Bergo, P. R. Salvador Junior, A. G. López, J. C. P. Alva, M. A. A. Gamba, F. G. Padilla-Padilla, A. E. B. Ruiz, J. Berlingieri, A. Bakbak, M. Gupta, K. Saunders, A. Costa-Vitali, P. R. Beaudry, R. Bhargava, Y. Khaykin, J. S. Healey, E. Crystal, Nadeau, Dhillon, Alistair Begg, C. Anderson, S. Baveja, D. Cross, A. Catanchin, D. Brieger, K. T. Lim, P. Davidson, R. Tan, R. Bhindi, J. Hickey, J. Layland, M. Bloch, C. Itty, B. Singh, P. Carroll, A. Lee, G. Starmer, R. Lehman

**Affiliations:** 1 Thrombosis Research Unit, Department of Medicine I, Division Haematology, University Hospital “Carl Gustav Carus” Dresden, Dresden, Germany; 2 Kings Thrombosis Service, Department of Haematology, Kings College London, London, UK; 3 Cardiology Clinical Academic Group, Molecular and Clinical Sciences Institute, St. George’s University of London, London, UK; 4 Centre for Cardiovascular Science, University of Edinburgh, Edinburgh, UK; 5 Georges Pompidou Hospital, René Descartes University, Paris, France; 6 Formerly Klinikum rechts der Isar, Technical University of Munich, Munich, Germany; 7 McMaster University, Hamilton, Canada; 8 Thrombosis Research Institute, London, UK; 9 University College of London, London, UK

**Keywords:** Anticoagulant, Antithrombotic, Atrial fibrillation, Outcomes, Registry, Rivaroxaban

## Abstract

**Background:**

Real-world data on non-vitamin K oral anticoagulants (NOACs) are essential in determining whether evidence from randomised controlled clinical trials translate into meaningful clinical benefits for patients in everyday practice. RIVER (RIVaroxaban Evaluation in Real life setting) is an ongoing international, prospective registry of patients with newly diagnosed non-valvular atrial fibrillation (NVAF) and at least one investigator-determined risk factor for stroke who received rivaroxaban as an initial treatment for the prevention of thromboembolic stroke. The aim of this paper is to describe the design of the RIVER registry and baseline characteristics of patients with newly diagnosed NVAF who received rivaroxaban as an initial treatment.

**Methods and results:**

Between January 2014 and June 2017, RIVER investigators recruited 5072 patients at 309 centres in 17 countries. The aim was to enroll consecutive patients at sites where rivaroxaban was already routinely prescribed for stroke prevention. Each patient is being followed up prospectively for a minimum of 2-years. The registry will capture data on the rate and nature of all thromboembolic events (stroke / systemic embolism), bleeding complications, all-cause mortality and other major cardiovascular events as they occur. Data quality is assured through a combination of remote electronic monitoring and onsite monitoring (including source data verification in 10% of cases). Patients were mostly enrolled by cardiologists (*n* = 3776, 74.6%), by internal medicine specialists 14.2% (*n* = 718) and by primary care/general practice physicians 8.2% (*n* = 417). The mean (SD) age of the population was 69.5 (11.0) years, 44.3% were women. Mean (SD) CHADS_2_ score was 1.9 (1.2) and CHA_2_DS_2_-VASc scores was 3.2 (1.6). Almost all patients (98.5%) were prescribed with once daily dose of rivaroxaban, most commonly 20 mg (76.5%) and 15 mg (20.0%) as their initial treatment; 17.9% of patients received concomitant antiplatelet therapy. Most patients enrolled in RIVER met the recommended threshold for AC therapy (86.6% for 2012 ESC Guidelines, and 79.8% of patients according to 2016 ESC Guidelines).

**Conclusions:**

The RIVER prospective registry will expand our knowledge of how rivaroxaban is prescribed in everyday practice and whether evidence from clinical trials can be translated to the broader cross-section of patients in the real world.

**Trial registration:**

Unique identifier: NCT02444221. Registerd 14 May 2015; Retrospectively Registered.

**Electronic supplementary material:**

The online version of this article (10.1186/s12959-019-0195-7) contains supplementary material, which is available to authorized users.

## Introduction

Atrial fibrillation (AF) is the most common sustained arrhythmia reported in adult patients [1] and is associated with an at least a five-fold increase in the risk of stroke [2]. Vitamin K antagonists (VKAs) formed the cornerstone of thromboembolic prophylaxis in patients with AF for many years. The introduction of non-vitamin K anticoagulants (NOACs), dabigatran, rivaroxaban, apixaban and edoxaban, provided physicians with agents with comparative efficacy and reduced potential for bleeding compared with vitamin K antagonists, while removing the need for dose titration, periodic laboratory testing and dietary restrictions that are necessary with VKAs [3–5]. Evidence from phase III studies showed that rivaroxaban, one of several oral direct Factor Xa inhibitors, is noninferior to warfarin for the reduction of stroke or systemic embolism in patients with AF [6] and significantly reduces rates of intracranial and fatal haemorrhages, but not rates of bleeding overall. Based on these results, rivaroxaban is now licensed in more than 130 countries worldwide for stroke prevention in patients with AF.

Real-world studies (such as Global Anticoagulant Registry in the FIELD-AF [GARFIELD-AF] [7], ORBIT-AF I & II) [8] and GLORIA-AF [9, 10] have demonstrated that NOAC use is increasing while post-marketing surveillance (such as Xarelto® for Prevention of Stroke in Patients with Atrial Fibrillation [XANTUS]) [11] and national studies (such as the EXPAND) [12, 13] have provided data on the safety and efficacy of rivaroxaban. However, most of these registries are disease-specific and evaluate treatment patterns and outcomes across different treatments. Therefore, additional drug-specific observational studies are needed to assess the clinical and health economic risk-benefits of specific NOACs and in particular, the factors that could potentially influence outcomes (e.g. compliance with prescribing guidelines, persistence and comorbidities) for each treatment alternative separately. This paper describes the design of the RIVaroxaban Evaluation in Real life setting (RIVER) registry and baseline characteristics of patients who received rivaroxaban as part of routine care. This paper also includes an initial analysis of the doses of rivaroxaban used in everyday practice as well as a description of the use of rivaroxaban in combination with antiplatelets in different clinical scenarios, such as patients with renal impairment.

## Methods

### Registry design

RIVER (NCT02444221) is an ongoing international, multicentre, prospective registry of patients with newly diagnosed non-valvular AF and at least one investigator-determined risk factor for stroke. All patients in this study are initiated on an anticoagulant treatment with rivaroxaban at the start of the study, as part of routine care, for the prevention of thromboembolic stroke.

**Fig. 1:**
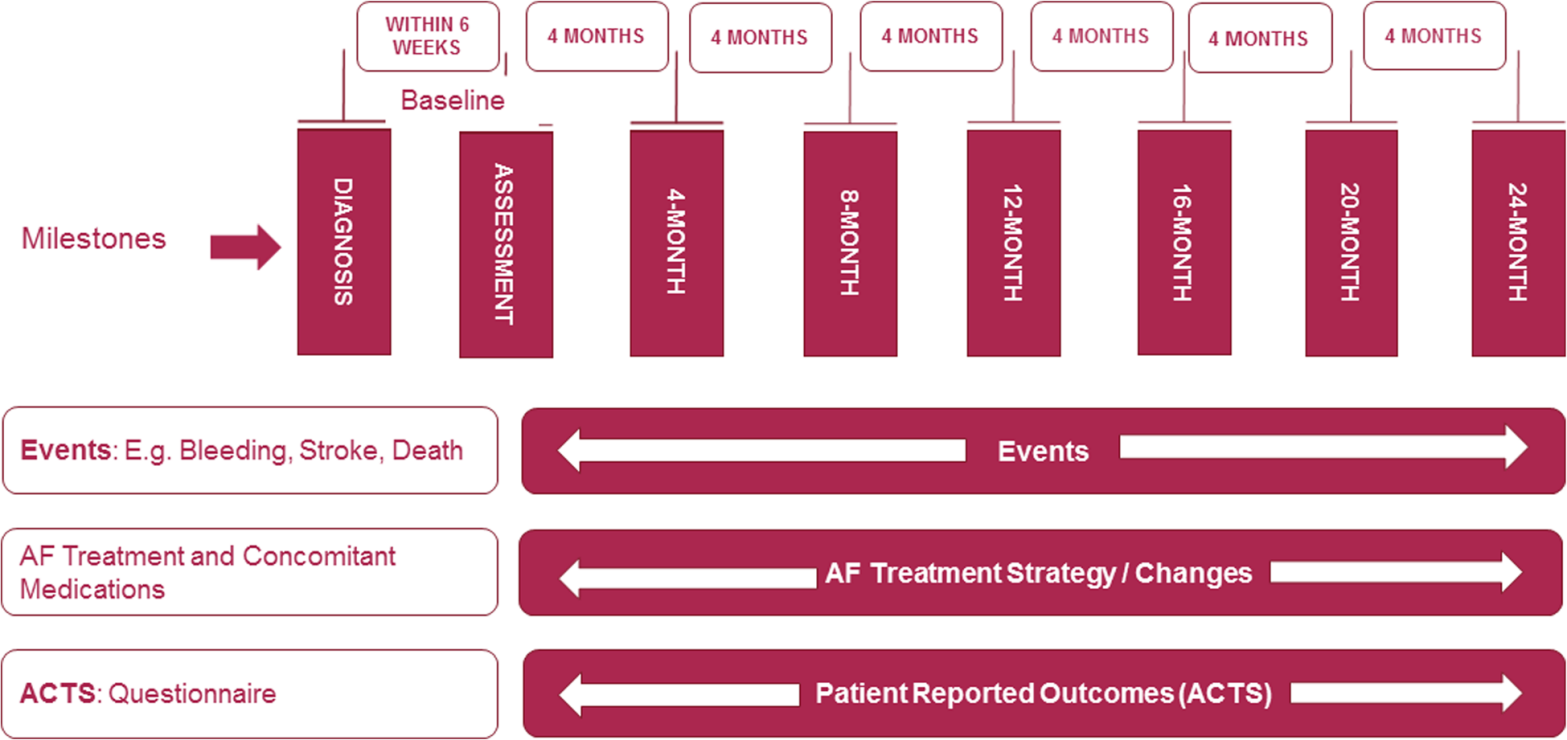
Cohort design and data collection

The goal of the RIVER registry is to provide insights into the clinical management and related outcomes of rivaroxaban-treated AF patients across a number of regions and across the spectrum of healthcare systems, in routine clinical management. Follow-up will be for a minimum of 2 years (and up to 3.5 years) after enrolment (Fig. 1). By capturing data from unselected patients treated in everyday practice, the registry has the potential to identify best practices as well as deficiencies in the use of rivaroxaban as an initial treatment for the prevention of thromboembolic events in patients with newly diagnosed AF. The decision to initiate treatment with rivaroxaban was based on clinicians’ judgement. The study was designed such that the collection of data would not interfere with the clinical management of patients or with the prescribing behaviours of attending physicians. No specific treatments, tests, or procedures were mandated or withheld from patients, and patients were free to withdraw from the registry at any time. The registry will not only assess the rates of clinical outcomes such as stroke and systemic embolization (SE), all-cause mortality, other major cardiovascular events (Table 1) and bleeding complications, but will also evaluate therapy persistence (including discontinuation, interruption and changes to the treatment regimen). Patients’ experiences using antithrombotic treatment will also be evaluated by the Anti-Clot Treatment Scale (ACTS) questionnaire.

**Table 1:** Main Outcome Measures in the RIVER Registry

Outcome	Category
Clinical events	Stroke (ischaemic and haemorrhagic)
	Transient ischaemic attack
	Peripheral/non-central nervous system embolism
	Pulmonary embolism
	Heart failure
	Myocardial infarction
	Hospitalization
	Sudden cardiac death
	Non-cardiovascular death
Bleeding events	
Severity	Major
	Non-major clinically relevant
	Minor
Location	Intracranial
	Ears, nose, throat
	Gastrointestinal
	Genitourinary
	Vascular access site
Outcome	Recovered
	Permanently disabled
	Fatal
Healthcare utilization used for bleeding event	Hospitalization
	Emergency room visit
	Surgery for bleeding
	Transfusion
	Physician consultation
Therapy persistence	Rate of discontinuation
	Duration of time on therapy
	Reasons for discontinuation
Hospitalization for any event	
Any other hospital visits	Inpatient, outpatient, and emergency room
Patients treated with vitamin K antagonists	Frequency and timing of monitoring
	INR recordings in relation to therapeutic range
	Location of testing (self-monitoring, general practitioner clinic, anticoagulant clinic, etc.)
	Dose adjustments
	Use of bridging anticoagulation necessitated by interruption of vitamin-K antagonist
	Outcomes in relation to INR fluctuation
Patients treated with additional antithrombotic therapy	Therapy changes (discontinuation, temporary interruptions and use of bridging therapy)
	Reasons of therapy changes (if applicable)
	Patient treatment satisfaction using the Anti-Clot Treatment Scale (ACTS), depending on the cohort and country (at 4, 12, 24 months)

*INR* International normalise ratio

Independent ethics committee and hospital-based institutional review board approvals were obtained, as necessary, for the registry protocol. The registry is being conducted in accordance with the Declaration of Helsinki, local regulatory requirements, and the International Conference on Harmonisation–Good Pharmacoepidemiological and Clinical Practice guidelines.

RIVER is an independent academic research initiative, sponsored by the Thrombosis Research Institute, London, UK and funded by an unrestricted research grant from Bayer AG, Berlin, Germany. The authors are responsible for the design and conduct of this study, data analyses and the final content of this paper.

### Registry population and site selection

The study population consists of consecutive patients enrolled prospectively at sites where rivaroxaban was already routinely prescribed for stroke prevention before the start of the study. Patients, receiving rivaroxaban as part of usual care, were eligible for enrolment in RIVER if they were 18 years of age or older, had a new diagnosis of non-valvular AF within the past 6 weeks and at least one additional risk factor for stroke as identified by the investigator. These risk factors were identified by clinicians and were not restricted to those in stroke prevention guidelines. Patients with a transient reversible cause of AF, those for whom follow-up is not foreseen or possible and all patients participating in interventional studies were excluded. All patients provided written informed consent to participate. Sites were chosen to be representative of AF care settings and included patients from multiple settings: office-based specialists, hospital departments (neurology, cardiology, geriatrics, internal medicine, and emergency room), anticoagulant clinics and general or family practice settings.

### Data capture

Patient visits are not mandated, but data collection using the electronic case report form (eCRF) occurs at 4-monthly intervals and captures all relevant data from the patients’ medical notes (Fig. 1).

A summary of the evaluations performed at baseline and at follow-up visits are outlined in Additional file 1: Table S1. At entry into the study, data are collected on the care setting, patients’ demographics, vital signs, medical history, the nature of AF (paroxysmal versus persistent versus permanent), symptoms of AF, relevant medications, including antiplatelet and other concomitant therapy and investigator-identified risk factors for stroke. The results from investigations at diagnosis (including ECG morphology, left ventricular ejection fraction [LVEF] measurement, full blood count, haemoglobin, platelet count and creatinine) are recorded as well as the treatment strategy for AF (rate or rhythm control) and the date and outcome following cardioversion. Information is collected on initial rivaroxaban dosing regimen, start and stop dates, changes in therapy, compliance concerns and the reason for suspending or terminating therapy sooner than intended (such as bleeding, patient decision, and/or physician decision). Patients’ experiences using antithrombotic treatment are recorded on the Anti-Clot Treatment Scale (ACTS) questionnaire at 4, 12 and 24 months.

At 4-monthly intervals, all routinely performed tests (including INR, haemoglobin, platelet count and creatinine), vital signs as well as all major events (stroke/transient ischaemic attack [TIA], bleeding / site of bleeding, death, myocardial infarction [MI], acute coronary syndrome [ACS], peripheral embolism) and hospitalizations/medical consultations are documented. For patients who switch to VKAs, data are also collected on INR, INR frequency and outcome related to INR fluctuation.

Based on the data collected from the eCRF, healthcare resource consumption will be captured so that the economic burden of AF can be computed both overall and per patient per year from the perspective of the payer, e.g. national health service, public/private/statutory insurance etc.

### Data management

Data are captured using an electronic case report form (eCRF) designed by eClinicalHealth Services, Stirling, UK and submitted electronically via a secure website to the registry-coordinating centre at the Thrombosis Research Institute who were responsible for checking the completeness and accuracy of data collected from medical records. All patients are assigned a unique identifier and personal identifiable data will be removed at the hospital source, ensuring anonymity and protecting confidentiality.

Data quality is assured through a combination of remote electronic monitoring and more conventional onsite monitoring (including source data verification in 10% of all cases), based on the validated quality assurance programmes developed in GARFIELD-AF [14]. The milestones for study eCRFs will be examined by the registry-coordinating centre at TRI, to ascertain completeness, accuracy and data queries sent to participating sites.

### Statistical analysis

Continuous variables are expressed as mean ± standard deviation (SD). Categorical variables are expressed as frequencies and percentages. Statistical analysis was performed using SAS software version 9.4 (SAS Institute Inc., Cary, NC, USA).

## Results

### Baseline characteristics

**Fig. 2:**
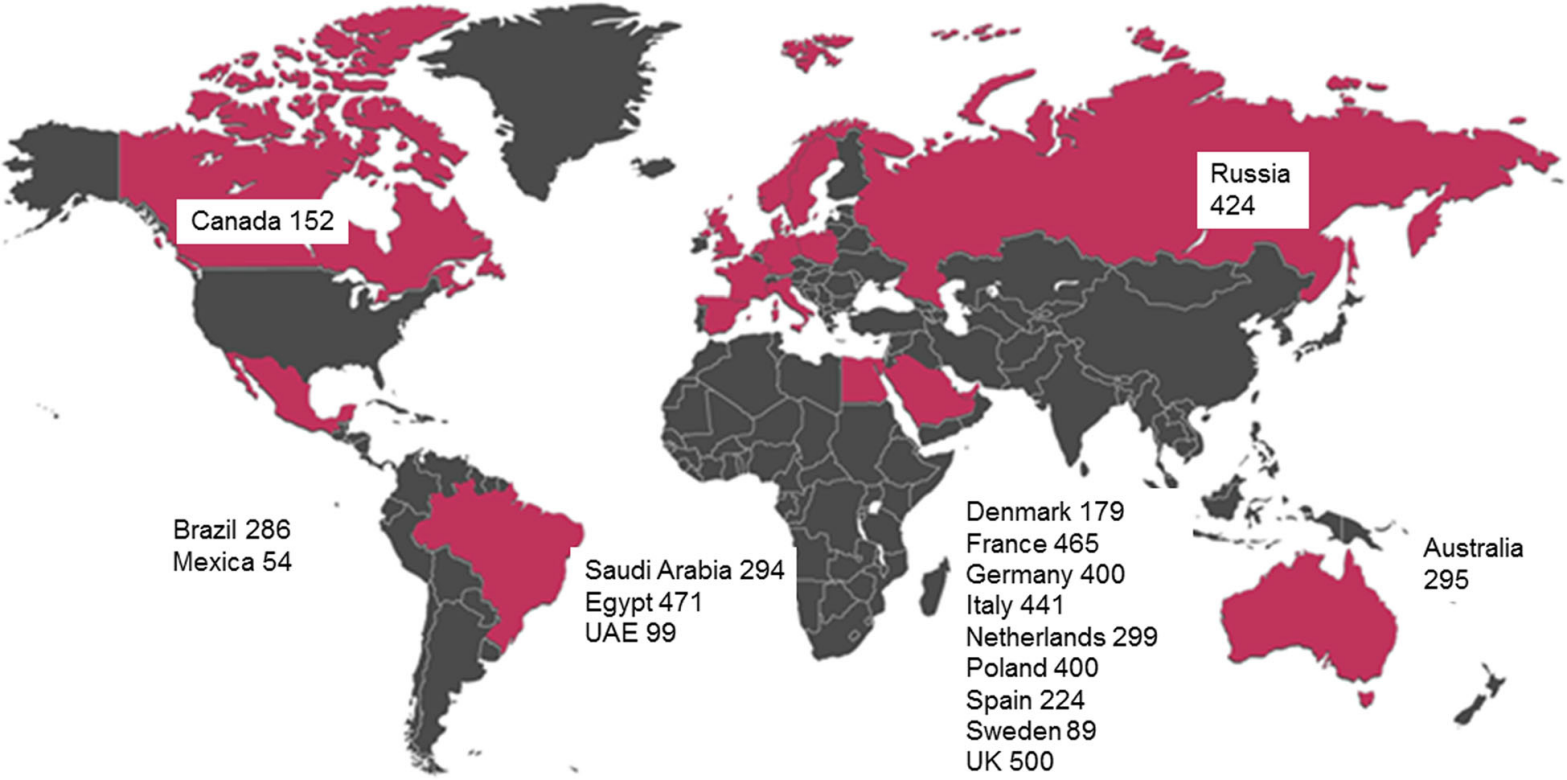
Location of enrolling centres of the RIVER Registry. Australia, Brazil, Canada, Denmark, Egypt, France, Germany, Italy, Mexico, Netherlands, Poland, Russia, Saudi Arabia, Spain, Sweden, UAE, UK

A total of 5282 patients were screened for inclusion into RIVER between January 2014 and June 2017 and 5072 eligible patients were included in the analysis of baseline characteristics. Reasons for the exclusion of 210 patients after screening were: age ≤ 18 years (*n* = 4), alternative AC treatment before rivaroxaban after AF diagnosis (*n* = 68), no risk factor for stroke (*n* = 22), patients having transient AF secondary to a reversible cause (*n* = 10), difficulty in long-term follow-up (*n* = 24), patients participating in other interventional studies (*n* = 9) and, finally, patients whose enrolment was not confirmed due to either patient’ decision (*n* = 27), death before consent (*n* = 1) or other reason (*n* = 77). Patients were recruited from 309 centres in 17 countries, including patients from Europe (67.5%) (Denmark, France, Germany, Italy, Netherlands, Poland, Russia, Spain, Sweden and UK), the Middle East (17.0%) (Egypt, Saudi Arabia and UAE), Australia (5.8%), Canada (3.0%), Brazil (5.6%) and Mexico (1.1%) (Fig. 2). Overall, 74.6% (*n* = 3779) of patients were enrolled by cardiologists, 14.2% (*n* = 718) by internal medicine specialists and 8.2% (*n* = 417) by primary care/general practice physicians.

Approximately one-third of patients (36.3%) in RIVER had asymptomatic AF. Among patients with symptomatic AF, the most common symptoms identified were: palpitations (38.4%), shortness of breath (31.8%) and tiredness (14.8%). At the time of enrolment, 34.2% of patients had new or unclassified AF, 34.2% paroxysmal AF, 15.9% persistent AF and 15.7% permanent AF.

Baseline patient characteristics are described in Table 2. Caucasian patients represented the largest proportion of patients in RIVER (80.3%), followed by Asians (5.6%) and hispanic/latino (3.7%). Beyond the factors defined by CHA_2_DS_2_-VASc, physicians also considered the following as risk factors for stroke: smoking (12.8%), obesity (19.5%), renal impairment (5.9%), proteinuria (1.9%), enlarged left atrium (16.4%), thyroid disease (5.9%), thrombus in atrium (0.4%) and Chagas disease (0.3%) amongst others (5.3%).

**Table 2:** Patient baseline characteristics

Variable	All patients (*N* = 5072)
Age^a^, mean ± SD, years	69.5 ± 11.0
Age group^a^, n (%)	
< 65	1444 (28.5)
65–74	1861 (36.8)
≥ 75	1758 (34.7)
Women, n (%)	2248 (44.3)
Body mass index^b^, mean ± SD, kg/m^2^	29.2 ± 5.7
Smoking status (current/previous)^c^, n (%)	1599 (35.3)
Pulse^d^, mean ± SD, bpm	85.1 ± 23.8
Medical history, n (%)	
Hypertension^e^	3899 (77.0)
Hypercholesterolemia^f^	2208 (43.9)
Diabetes mellitus^g^	1227 (24.3)
Congestive heart failure^h^	1086 (21.5)
Family history of cardiac disease^i^	618 (19.5)
Coronary artery disease^j^	967 (19.2)
Current/History of myocardial infarction or unstable angina^k^	718 (14.2)
Chronic renal disease^l^	
Mild renal dysfunction (stage: I, II)	629 (13.5)
Moderate renal dysfunction (stage: III)	186 (4.0)
Severe renal dysfunction (stage: IV, V)	26 (0.6)
Left ventricular ejection fraction < 40%^m^	305 (9.5)
Stroke^n^	365 (7.2)
Transient ischaemic attack^o^	331 (6.5)
Aortic or peripheral artery disease^p^	252 (5.0)
Carotid disease^q^	212 (4.2)
History of bleeding^r^	118 (2.3)
Heavy alcohol consumption^s^	83 (2.1)
Venous thromboembolism^t^	103 (2.0)
Systemic embolization^u^	56 (1.1)
Cirrhosis^v^	20 (0.4)

^a^Data not available for 9 patients

^b^Data not available for 586 patients

^c^Data not available for 541 patients

^d^Data not available for 236 patients

^e^Data not available for 9 patients

^f^Data not available for 44 patients

^g^Data not available for 12 patients

^h^Data not available for 10 patients

^i^Data not available for 1906 patients

^j^Data not available for 28 patients

^k^Data not available for 21 patients

^l^Data not available for 395 patients

^m^Data not available for 1870 patients

^n^Data not available for 11 patients

^o^Data not available for 15 patients

^p^Data not available for 29 patients

^q^Data not available for 53 patients

^r^Data not available for 12 patients

^s^Data not available for 1087 patients

^t^Data not available for 15 patients

^u^Data not available for 17 patients

^v^Data not available for 24 patients

**Fig. 3:**
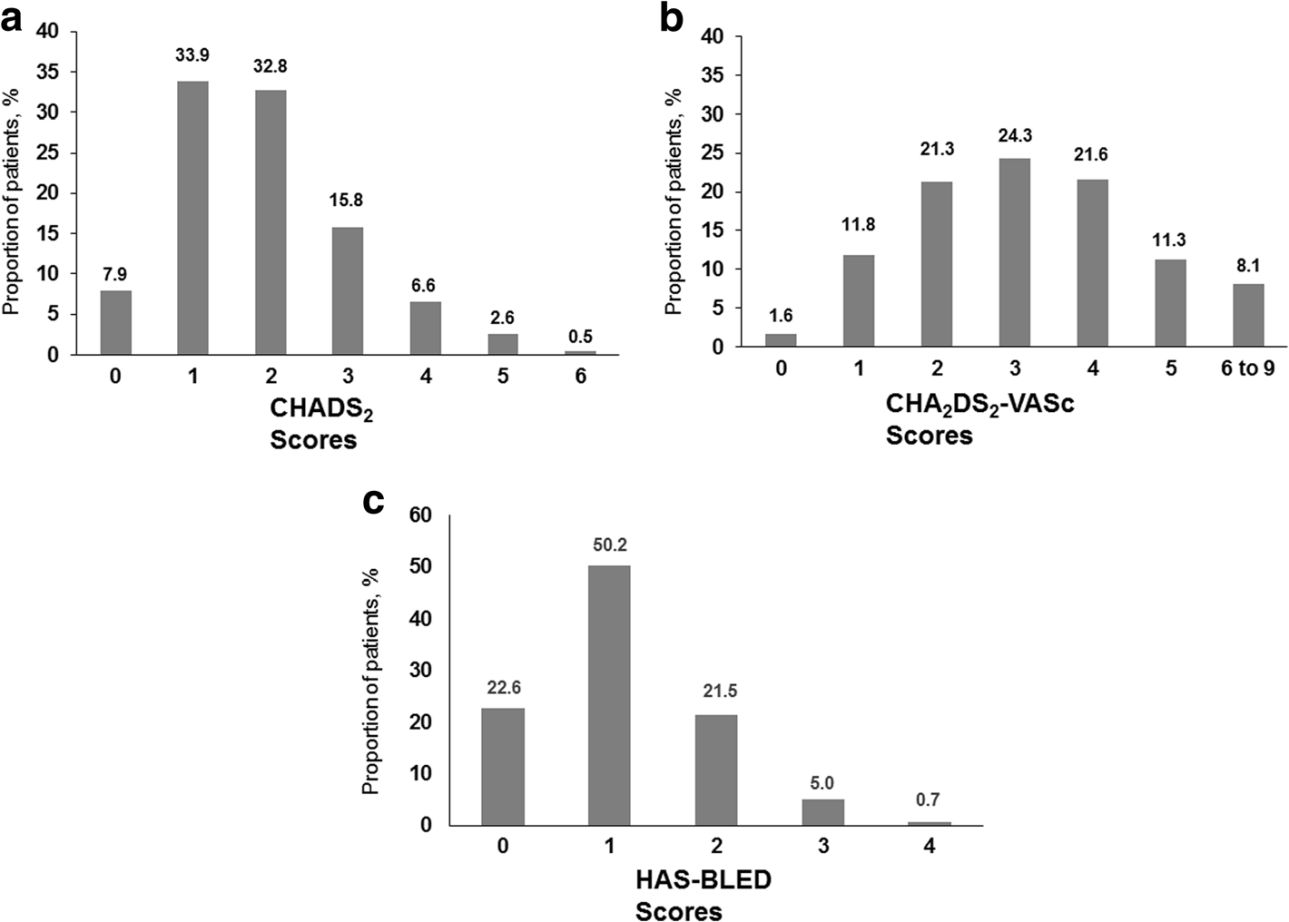
**a** Distribution of CHADS_2_ **b** CHA_2_DS_2_-VASc and **c** HAS-BLED scores in RIVER registry

Figure 3 describes the prescribing of rivaroxaban according to the post-hoc calculation of CHADS_2_ and CHA_2_DS_2_-VASc risk scores. The mean (SD) CHADS_2_ score for patients in the RIVER was 1.9 (1.2) and 58.2% of patients had a score of ≥2. Mean (SD) CHA_2_DS_2_-VASc score was 3.2 (1.6). Most patients met the recommended threshold for AC therapy (86.6% for 2012 ESC Guidelines, and 79.8% of patients according to 2016 ESC Guidelines). The mean (SD) HAS-BLED score was 1.1 (0.8) and the most commonly observed bleeding risk factors were: hypertension (77.0%), stroke (7.2%) and moderate-to-severe chronic renal disease (4.6%).

### Rivaroxaban dosing

Almost all patients (98.5%) were prescribed a single daily dose of rivaroxaban, most commonly 20 mg (76.5%) and 15 mg (20.0%), while10 mg dose was prescribed in only 2.1% of patients. Creatinine clearance (CrCl) information at enrolment, estimated using the Cockcroft-Gault equation, was available for 3135 of 5072 (61.8%) patients. The mean (SD) CrCl was 83.7 (35.6) mL/min and 86.1% patients had CrCl ≥50 mL/min. Among patients with normal or mild renal function (CrCl ≥50 mL/min), 2246 (83.2%) were prescribed the recommended rivaroxaban dose of 20 mg once daily at baseline. Of patients with moderate or severe renal impairment (CrCl 15–49 mL/min), 246 (56.6%) patients received rivaroxaban 15 mg once daily, 173 (39.8%) 20 mg once daily and 16 (3.7%) 10 mg once daily as their initial treatment.

### Concomitant antiplatelet therapy

Just under one in five (17.9%, *n* = 904) patients who were initiated on rivaroxaban at the time of diagnosis of AF also received concomitant antiplatelet therapy. Use of antiplatelets was higher in patients with a CHA_2_DS_2_-VASc score of 2–9 (19.4%) than in those with a score of 0 or 1 (9.2%) (Table 3); 229 (25.3%) had a coronary artery disease (CAD), peripheral artery disease (PAD) or carotid disease. The most frequently used antiplatelet was aspirin in 55.4% (501 of 904) of cases. Overall, 76 (1.5%) patients received dual AP therapy (ADP receptor/P2Y12 and aspirin).

**Table 3:** Use of antithrombotic drugs at diagnosis, overall and according to CHA_2_DS_2_-VASc scores of 0 or 1 and 2–9

Antiplatelet drug^a^	All patients (*N* = 5072)	Patients with CHA_2_DS_2_-VASc score of 0 or 1 (*N* = 665)	Patients with CHA_2_DS_2_-VASc score 2–9 (*N* = 4289)
Acetylsalicylic acid (Aspirin/ASA)	501 (9.9)	28 (4.2)	465 (10.8)
ADP receptor/P2Y12 inhibitor	248 (4.9)	13 (2.0)	231 (5.4)
Glycoprotein IIb/IIIa inhibitor	9 (0.2)	2 (0.3)	6 (0.1)
Prostaglandin analogue	71 (1.4)	5 (0.8)	66 (1.5)
Other antiplatelet	8 (0.2)	2 (0.3)	6 (0.1)

^a^Categories are not mutually exclusive

## Discussion

Observational or non-interventional studies play an important role in understanding and confirming the clinical effectiveness of interventions across the spectrum patients treated in routine practice.

RIVER is an independent treatment registry of patients from multiple care settings and patients were recruited from 17 countries. The RIVER registry provides an opportunity to examine the impact of different recruitment methodologies on the characteristics and treatment between the registries. RIVER will document the regional heterogeneity in the clinical presentation and assess the rate of stroke and systemic embolism and other outcomes with specific reference to the incidences of bleeding and therapy persistence. In addition to reporting on pre-specified clinical and economic outcomes, analyses from RIVER will be hypothesis generating, allowing the exploration of some aspects of the real world situation of AF treated with rivaroxaban and outcomes.

The degree of the patient involvement in the generation of data is expected to provide unique insights into the patients-reported outcomes. Patients’ experiences using antithrombotic treatment are recorded on the Anti-Clot Treatment Scale (ACTS) questionnaire. It is important to recognise that registries differ in their design, recruitment strategies, care settings, geographical spread and duration of follow-up. Compared to other ongoing prospective registries, the RIVER registry has the potential to capture the medicosocial burden due to AF in large-scale populations by employing broad inclusion and exclusion criteria in widely representative populations of patients with AF who are treated with rivaroxaban, across a range of clinical settings and to capture long-term follow-up data. The value of RIVER registry is enhanced by a quality assurance programme and the supervision of an independent Audit Committee, which oversees site-dependent verification, remote site monitoring and electronic database monitoring to ensure data quality.

There are several phase IV studies (such as XANTUS [15], XAPASS [16], XASSURE (China; NCT02784717) and registries such as EXPAND [12]) are ongoing to investigate rivaroxaban in patients with NVAF in the real world clinical settings. The XANTUS program comprises three studies: the XANTUS (Europe, Israel plus enrollment in Canada; NCT01606995), XANTUS-EL (Eastern Europe, Eastern Mediterranean, Middle East, Latin America; NCT01800006) and XANAP (Asia-Pacific; NCT01750788) [15].

Other ongoing non-interventional registries also provide real-world data on the effectiveness and safety of rivaroxaban, including the Global Anticoagulant Registry in the FIELD (GARFIELD)-AF [17], Outcomes Registry for Better Informed Treatment of Atrial Fibrillation (ORBIT)-AF I and ORBIT-AF II [18–21], the Global Registry on Long-Term Oral Anthithrombotic Treatment in pateints with Atrial Fribrillation (GLORIA-AF) [22–26] and the Dresden NOAC Registry [4, 5, 27] (Additional file 1: Table S2).

In the presence of all these observational studies, it is important to highlight that there are a number of important differences in the design (Additional file 1: Table S2) and recruitment of patients (Table 4) in RIVER compared with these registries. RIVER is a drug-specific registry which allows studying of drug utilisation patterns including off-label use whereas other registries like GARFIELD-AF, ORBIT-AF I and II, GLORIA-AF and Dresden NOAC are disease-specific registries which allows studying of burden, management and adverse event profile for different treatments related to a specific disease. Patients in XANTUS study were enrolled from Europe, Israel and Canada whereas EXPAND and XAPASS are Japanese registries and ORBIT-AF registries are restricted to USA [19]. By contrast, RIVER has recruited patients more broadly from 17 countries (outside Asia) including Europe, the Middle East, Latin America, Canada and Australia. RIVER provides long-term follow-up data of 2 years where as XANTUS had 1-year follow-up data. RIVER is a more contemporary population in which physicians’ now have experience using the NOACs. It is a known phenomenon that, after approval of new treatments, physicians start to apply these first in well-defined, carefully selected populations and expand it to other populations only after a period of time, which is reflecting learning curves based on increased comfort levels, communication of real world evidence, newly reported and previously unknown side effects or changes in guideline recommendations [28–30] . As a consequence, treatment patterns and outcomes may shift over time, which is an additional justification for more contemporary registries in SPAF. All of these factors may have contributed to the observed differences in patients characteristics, compared to other observational studies. Patients in RIVER are younger (mean age: 69.5 years) than patients in EXPAND (72 yrs), XANTUS (71.5 yrs), and XAPASS (73.1 yrs). The mean BMI of patients in RIVER (29.2 ± 5.7) is higher than the Japanese cohort in XAPASS (23.7 ± 3.8) (Table 4). Patients studied in RIVER also had a lower risk of stroke, with a mean CHADS_2_ scores of 1.9 and 13.4% had a prior episode of stroke/TIA/SE when compared to XANTUS (2.0, 19%) and XAPASS (2.2; 23.7%) (Additional file 1: Table S3) and other NOAC trials, such as RE-LY (2.1; 20.0%) and ARISTOTLE (2.1; 19.4%) [31, 32]. Nevertheless, patients in RIVER had high rates of comorbid conditions, including hypertension, hypercholesterolemia, diabetes mellitus, heart failure and coronary artery disease. However, the proportion of patients (8.6%) with renal dysfunction (CrCl < 50 mL/min) in RIVER was similar to XANTUS (9.4%) and lower than Japanese cohorts in EXPAND (20.8%) and XAPASS (23.9%); although this observation may be due to the higher rates of missing CrCl measurements in both RIVER (38.0%) and XANTUS (34.4%). Patients in RIVER and XANTUS received higher doses of rivaroxaban (20 mg/day [76.5 and 78.7% patients, respectively], 15 mg/day [20.0 and 20.8% patients, respectively]) than those in EXPAND (15 mg/day [56.6% patients], 10 mg/day [43.4% patients]), suggesting differences in the guideline recommendations, which are based on patient characteristics and ethnicities. In Japan, the regular dosage of rivaroxaban is 15 mg od, which is lower than the global recommended dosage of 20 mg od [33].

**Table 4:** Comparison of adjusted demographic and baseline clinical characteristics of patients from the international observational studies (RIVER and XANTUS), studies from Japan (EXPAND and XAPASS)

	RIVER	XANTUS	EXPAND	XAPASS
Patients, n	5072	6784	7141	11,308
Patient characteristics				
Sex, male (%)	55.7	59.2	67.7	61.9
Age, mean (SD)	69.5 (11.0)	71.5 (10.0)	71.6 (9.4)	73.1 (9.9)
BMI (Kg/m^2^), mean (SD)	29.2 (5.7)	28.3 (5.0)	NA	23.7 (3.8)
Creatinine clearance (mL/min), mean (SD)	83.7 (35.6)	NA	69.7 (26.2)	67.7 (28.9)
< 15 mL/min (%)	0.02	0.3	NA	0.03
≥ 15 to < 30 mL/min (%)	0.7	1.1	1.9	2.8
≥ 30 to < 50 mL/min (%)	7.9	8.0	18.9	21.1
≥ 50 mL/min (%)	53.4	56.2	74.6	68.0
Missing (%)	38.0	34.4	4.7	8.2
Rivaroxaban dose/dosing frequency				
10 mg once daily	105 (2.1)	NA	NA	NA
15 mg once daily	1008 (20.0)	1410 (20.8)	NA	NA
20 mg once daily	3867 (76.5)	5336 (78.7)	NA	NA
10 mg twice daily	10 (0.2)	NA	NA	NA
15 mg twice daily	29 (0.6)	NA	NA	NA
20 mg twice daily	22 (0.4)	NA	NA	NA
10/15/20 mg other dosing Regimen	12 (0.2)	35 (0.5)	NA	NA
CHADS_2_ score, mean (SD)	1.9 (1.2)	2.0 (1.3)	2.1 (1.3)	2.2 (1.3)
CHA_2_DS_2_-VASc score, mean (SD)	3.2 (1.6)	3.4 (1.7)	3.4 (1.7)	NA
Comorbidity/medical history				
Congestive heart failure (%)	21.5	18.6	26.1	25.0
Hypertension (%)	77.0	74.7	70.9	74.3
Diabetes mellitus (%)	24.3	19.6	24.3	22.3
History of stroke/systemic embolism/TIA (%)	13.4	19.0	24.1	23.7
Type of AF				
Paroxysmal (%)	34.2	40.7	44.8	NA
Non-paroxysmal (persistent/permanent) (%)	31.6	40.8	55.2	NA
First diagnosed (%)	34.2	18.5	NA	NA

## Limitations

Centres for recruitment of patients in RIVER are limited to the care settings where rivaroxaban is the standard of care and includes sites beyond those included in the clinical trials. Comparison of different treatments are not possible, as there is no comparator drug, while larger prospective disease registries such as GARFIELD-AF and drug registries such as GLORIA-AF and ORBIT-II have the potential to conduct comparative effectiveness studies between different treatment approaches in newly diagnosed patients with AF. Due to the observational design of the study, laboratory and other investigations could not mandated.

## Strengths

RIVER is an independent academic research initiative. This prospective registry was designed with a novel approach to outcomes research, including the following features: sites representation of national AF care settings, randomised site selection, unselected eligible patients enrolled consecutively with a follow-up period of minimum 2 years and extensive monitoring and audit including source data verification of 10% of all eCRFs. Like other prospective registries, such as GARFIELD-AF [34], RIVER aims to overcome the inherent bias of retrospective cohort studies from national registries and claims databases [35–39].

## Conclusion

The RIVER prospective registry will expand our knowledge of how rivaroxaban is prescribed in everyday practice and whether evidence from clinical trials can be translated to the broader cross-section of patients treated in the real world.

## Additional file

Additional file 1: **Table S1.** Evaluations performed at Baseline and at Follow-Up Visits. **Table S2.** Study design of ongoing real-world studies of rivaroxaban. **Table S3.** Comparison of distribution of baseline CHADS_2_, CHA_2_DS_2_-VASc or HAS-BLED scores of patients from observational RIVER, XANTUS, EXPAND and XAPASS studies. (DOC 155 kb)

## Abbreviations

AC: Anticoagulant
ACTS: Anti-Clot Treatment Scale
AF: Atrial fibrillation
AP: Antiplatelet
CAD: Coronary artery disease
CHF: Congestive heart failure
CrCl: Creatinine clearance
LVEF: Left ventricular ejection fraction
MI: Myocardial infarction
NOACs: Non-vitamin K anticoagulants
NVAF: Non-valvular atrial fibrillation
PAD: Peripheral artery disease
SE: Systemic embolism
TIA: Transient ischaemic attack
VKA: Vitamin K antagonist
