# Supplementary material for: International longitudinal registry of patients with atrial fibrillation and treated with rivaroxaban: RIVaroxaban Evaluation in Real life setting (RIVER)
Source: Thromb J. 2019 Apr 25;17:7. doi: 10.1186/s12959-019-0195-7 (PMC6482585; doi:10.1186/s12959-019-0195-7)
Supplement: Supplementary file 1 — Table S1. Evaluations performed at Baseline and at Follow-Up Visits. Table S2. Study design of ongoing real-world studies of rivaroxaban. Table S3. Comparison of distribution of baseline CHADS2, CHA2DS2-VASc or HAS-BLED scores of patients from observational RIVER, XANTUS, EXPAND and XAPASS studies. (DOC 155 kb) [file 12959_2019_195_MOESM1_ESM.doc]

**International Longitudinal Registry of Patients with Atrial Fibrillation and Treated with Rivaroxaban: RIVaroxaban Evaluation in Real Life setting (RIVER)**

Jan Beyer-Westendorf^1,2^, A. John Camm^3^, Keith A. A. Fox^4^, Jean-Yves Le Heuzey^5^, Sylvia Haas^6^, Alexander G.G. Turpie^7^, Saverio Virdone^8^, Ajay K. Kakkar^8, 9^ for the RIVER Registry Investigators*****

**Table S1**. **Evaluations performed at Baseline and at Follow-Up Visits**

| **Baseline visit** | **Follow-up visits** |
| --- | --- |
| Demographics and baseline characteristics (age, sex, race, height, weight, blood pressure status, pulse)  Medical history, including stroke/TIA, carotid surgery/stenting, coronary stenting (bare metal or drug eluting), coronary surgery, myocardial infarction, peripheral vascular disease/stenting/surgery, possible bleeding risk factors, possible therapy contraindications, cardiovascular risk factors  Nature of atrial fibrillation  Date of atrial fibrillation diagnosis  Method of atrial fibrillation diagnosis  Arrhythmia-related symptoms  Proposed atrial fibrillation treatment strategy  Drug therapy: cardiac, non-cardiac, including over-the-counter treatments, rate or rhythm control (electrocardioversion or pharmacological cardioversion)  Anticoagulant/antiplatelet therapy  Treatment decision (patient and physician)  Anti-Clot Treatment Scale, after antithrombotic therapy taken for 4 weeks; self-completed by patient, depending on country and cohort  Laboratory Tests  Red blood cells count, white blood cell count,  Platelet count, creatinine, hemoglobin,  Protein dip stick, | Occurrence of clinical events since previous visit  Healthcare utilization (hospitalization, emergency room, physician consultation, etc.)  Hospitalization (reasons for)  Unexpected medical consultation  International normalized ratio testing  Atrial fibrillation treatment  Stroke prophylaxis  For outcome events: type of event, method of diagnosis, treatment  Medical history update  Patient treatment satisfaction |

INR, international normalized ratio

**Table S2 : Study design of ongoing real-world studies of rivaroxaban**

| **Study name** | **Population size** | **Patient enrolment – key design features** | **Follow-up** |
| --- | --- | --- | --- |
| **Registries** | | | |
| GARFIELD-AF | Target: 57,000  To date: 57,262  Estimated last follow-up: 2019 | Enrolment: Adults (≥18 years) with newly diagnosed AF and ≥1 investigator-defined stroke risk factor in five sequential cohorts.  Setting: Approximately 1000 sites in 35 countries from nationally representative clinical settings (hospital and community). Patients are managed according to local standard practice.  Endpoints: Describes change in treatment practice (including patients on VKA therapy and no thearpy). Quantify rates of stroke and systemic embolism, major bleeding and all-cause mortality. Assess therapy persistence (including discontinuation, interruption and change of regimen) and fluctuations in the international normalised ratio for patients on vitamin K antagonist. | ≥6 years |
| ORBIT-AF I | Target: 10,000  To date: 10,179 | Enrolment: Men and women aged 18 years or older with electrocardiographically documented AF.  Setting: Approximately 200 sites in US from different care settings.  Endpoints: Define current practice patterns for stroke prevention. Assess the adoption and impact of emerging antithrombotic and antiarrhythmetic therapies on outcomes in AF, including patient reported outcomes (PRO) and health care resource use. Quantify rates of stroke or non-central nervous system, systemic embolism, major bleeding and all-cause mortality. | ≥2 years |
| ORBIT-AF II | Target: 15,000 | Enrolment: Men and women aged 21 years or older with new-onset AF, electrocardiographically confirmed AF and those newly transitioned to NOACs (within the previous 3 months).  Setting: Approximately 300 sites in US from different care and clinical settings.  Endpoints: Evaluate the safety of NOACs, including factor Xa inhibitors and direct thrombin inhibitors, in outpatients with AF. Management of patients with AF undergoing cardiac procedures and their outcomes. Describe AF patient characteristics, with specific attention to the use of NOAC and high-risk subgroups, such as those with chronic kidney disease, acute coronary syndromes, or risk factors for stroke or bleeding. Incidence of major bleeding. | Every 6 months to 2 years |
| Dresden NOAC | Target: 4500 (incl. 1200 rivaroxaban SPAF patients) | Enrolment: Patients >18 years of age. Planned anticoagulation with any NOAC for at least 3 months. Therapeutic NOAC indication including AF, deep vein thrombosis (DVT), pulmonary embolism (PE) and other indications.  Setting: German registry, a network of >230 physicians from private practies and hospitals.  Endpoints: Composite endpoint of fatal or non-fatal major cardiovascular events (Acute coronory syndrome, stroke/ transient ischaemic attck or systemic embolism) and major bleeding. |  |
| EXPAND | To date: 7141 (Nov 2012-June 2014)  Last follow-up: March 2016 | Enrolment: Patients ≥20 years of age who were diagnosed with NVAF and using or about to use rivaroxaban were enrolled.  Setting: Japanese registry with 684 centers. Patients are treated according to local best practice.  Endpoints: Composite cumulative incidence of symptomatic stroke (ischemic or hemorrhagic) and SE, major and non-major bleeding events and all-cause death. | 4 years |
| Non-Interventional | | | |
| XANTUS | Target: 10,934  Estimated last follow-up: March 2015 | Enrolment: Men and women aged 18 years or older with nonvalvular AF who start rivaroxaban therapy.  Setting: Enrollment will take place at up to 300 sites, each of which should enroll a minimum of 20 and a maximum of 60 patients. Sites must be able to capture data electronically and provide long-term follow-up for patients.  Endpoints: Short- and long-term mortality, major and non-major bleeding events, Symptomatic thromboembolic events, Rates of AEs and serious AEs across patients with different baseline risk profiles for stroke or bleeding. | 1 years (For each patient enrolled) |
| XASSURE^a^ | Target: 6000 | Enrolment: Eligible patients comprise men or women with NVAF starting  rivaroxaban therapy to reduce the risk of stroke/SE.  Setting: Chinese registry. Decision regarding dose and duration of treatment made at the discretion of the attending investigator.  Endpoints: Major and non-major bleeding treatment persistence with rivaroxaban, and rates of AEs or serious AEs across patients with different baseline. |  |
| Post-Authorization Safety & Effectiveness study | | | |
| XAPASS | To date: 11,308 (June 2014)  Estimated last follow-up: 2019 | Enrolment: Eligible patients comprise men or women with NVAF starting  rivaroxaban therapy to reduce the risk of stroke/SE.  Setting: Japanese registry. Sites are locally representative of primary and secondary care settings. Patients managed according to local standard practice.  Endpoints: Assessment and estimation of the safety of rivaroxaban in routine clinical practice. All-cause mortality, Major and non-major bleeding treatment persistence with rivaroxaban, and rates of AEs or serious AEs across patients with different baseline risk profiles for stroke or bleeding. | 5 year |

a : https://clinicaltrials.gov/ct2/show/NCT02784717

**Table S3** . **Comparison of distribution of baseline CHADS_2_, CHA_2_DS_2_-VASc or HAS-BLED scores of patients from observational RIVER, XANTUS, EXPAND and XAPASS studies**.

|  | **RIVER** | **XANTUS** | **EXPAND** | **XAPASS** |
| --- | --- | --- | --- | --- |
| Patients, n | 5072 | 6784 | 7141 | 11,308 |
| CHADS_2_ score |  |  |  |  |
| 0 | 7.9 | 10.4 | 10.2 | 8.7 |
| 1 | 33.9 | 30.4 | 27.2 | 24.8 |
| 2 | 32.8 | 30.0 | 28.9 | 30.1 |
| 3 | 15.8 | 16.4 | 18.7 | 19.5 |
| 4 | 6.6 | 9.1 | 10.3 | 11.4 |
| 5 | 2.6 | 3.3 | 3.9 | 4.5 |
| 6 | 0.5 | 0.5 | 0.9 | 0.9 |
| CHA_2_DS_2_-VASc score |  |  |  |  |
| 0 | 1.6 | 2.6 | 3.1 | NA |
| 1 | 11.8 | 10.1 | 10.5 | NA |
| 2 | 21.3 | 19.4 | 18.4 | NA |
| 3 | 24.3 | 23.3 | 23.0 | NA |
| 4 | 21.6 | 20.7 | 20.7 | NA |
| 5 | 11.3 | 12.3 | 13.5 | NA |
| 6-9 | 8.1 | 11.6 | 10.7 | NA |
| HAS-BLED score |  |  |  |  |
| 0 | 22.6 | 4.6 | 12.0 | NA |
| 1 | 50.2 | 25.8 | 48.2 | NA |
| 2 | 21.5 | 41.9 | 28.6 | NA |
| 3 | 5.0 | 20.3 | 9.4 | NA |
| 4 | 0.7 | 5.6 | 1.7 | NA |
| 5 | NA | 1.2 | 0.1 | NA |
| 6-9 | NA | 0.2 | 0.0 | NA |
